# Elevated risk of cervical cancer in elderly women with incident ulcerative colitis in South Korea

**DOI:** 10.1038/s41598-023-33476-6

**Published:** 2023-05-23

**Authors:** Jihoon Kim, Halim Jo, Min Chul Ha, Hyunil Kim, Jung Kuk Lee, Jae Hun Han, San-Hui Lee, Dae Ryong Kang, Su Young Kim, Hyun-Soo Kim, Hee Man Kim

**Affiliations:** 1 Department of Physiology, Yonsei University Wonju College of Medicine, Wonju, Korea; 2 Department of Medicine, Yonsei University Wonju College of Medicine, Wonju, Korea; 3 Department of Internal Medicine, Yonsei University Wonju College of Medicine, Wonju, Korea; 4 Department of Biostatistics, Yonsei University Wonju College of Medicine, Wonju, Korea; 5 Department of Obstetrics and Gynecology, Yonsei University Wonju College of Medicine, Wonju, Korea; 6 Department of Precision Medicine and Biostatistics, Yonsei University Wonju College of Medicine, Wonju, Korea; 7 Health Promotion Center, Gangnam Severance Hospital, Yonsei University College of Medicine, Seoul, Korea

**Keywords:** Inflammatory bowel disease, Crohn's disease, Ulcerative colitis

## Abstract

The association between ulcerative colitis (UC) and uterine cervical cancer is still unclear. To investigate cervical cancer risk in South Korean women with UC, we analyzed the Korean National Health Insurance claims data. UC was defined using both ICD-10 codes and UC-specific prescriptions. We analyzed incident cases of UC diagnosed between 2006 and 2015. Age-matched women without UC (control group) were randomly selected from the general population (1:3 ratio). Hazard ratios were calculated using multivariate Cox proportional hazard regression, and the event was defined as occurrence of cervical cancer. A total of 12,632 women with UC and 36,797 women without UC were enrolled. The incidence of cervical cancer was 38.8 per 100,000 women per year in UC patients and 25.7 per 100,000 women per year in controls, respectively. The adjusted HR for cervical cancer was 1.56 (95% CI 0.97–2.50) in the UC group with reference to the control group. When stratified by age, the adjusted HR for cervical cancer was 3.65 (95% CI 1.54–8.66) in elderly UC patients (≥ 60 years) compared to elderly control group (≥ 60 years). Within UC patients, increased age (≥ 40 years) and low socioeconomic status were associated with an increased risk of cervical cancer. The incidence of cervical cancer was found to be higher among elderly patients (≥ 60 years) with newly diagnosed UC in South Korea, compared to age-matched controls. Therefore, regular cervical cancer screening is recommended for elderly patients who have recently been diagnosed with UC.

## Introduction

Ulcerative colitis (UC) is an chronic inflammatory disease of the large intestine that is mediated by the immune system. Prolonged UC is associated with an increased risk of intestinal cancers. In addition, some extraintestinal cancers and cervical cancer have been reported to be associated with UC^1–3^, although the association with cervical cancer remains controversial^4–6^. Cervical cancer is the fourth most common type of cancer and the fourth leading cause of cancer-related deaths in women worldwide^7^. It is caused by persistent infection with high-risk variants of the human papillomavirus (HPV) and is often seen in immunosuppressed patients with HIV/AIDS or organ transplantation^8^. While some studies have suggested an association between UC and cervical cancer, the evidence remains inconclusive, and further research is needed to clarify this relationship. Understanding the risk factors for cervical cancer in UC patients could help inform screening and prevention strategies, and ultimately improve outcomes for this population. Therefore, we conducted a population-based study to determine the risk of cervical cancer in patients newly diagnosed with UC.

## Methods

### Data source

Our study used data provided by the Korean National Health Insurance (NHI), which is a mandatory nationwide insurance system operated by the Korean government. The NHI database, which covers the entire population of South Korea (which stands at approximately 51 million as of 2019), contains information on all patients, including demographic characteristics, diagnosis, pharmaceutical prescriptions, procedures, and comorbidities defined according to the International Classification of Diseases, 10th revision (ICD-10)^3^.

### Patient identification

To ensure accurate diagnosis, we followed a specific process. Firstly, we selected patients diagnosed with UC between January 2004 and December 2015 from the NHI database, who had the requisite ICD-10 diagnostic code (K51.0–51.9 for UC) and a prescription for UC medication. Secondly, we used a 2-year washout period (January 2004–December 2005) to exclude prevalent cases among patients with UC, to avoid confounding incidence rate due to pre-existing diseases. Patients with pre-existing UC would have visited the hospital at least once within 2 years, so we considered new diagnoses only after the 2 years washout period. We defined the prescriptions for UC as the use of steroids for 3 months, 5-aminosalicylic acid, immunomodulators like azathioprine, 6-mercaptopurine, and/or methotrexate, and/or biologic agents like tumor necrosis factor-alpha antagonists at least once.

We analyzed incident cases of UC diagnosed between 2006 and 2015 among 957,056,482 beneficiaries of medical aid and NHI. To validate our diagnosis of IBD in Korea, we used a study that showed our definition had a sensitivity of 93.1 (91–94.7) and specificity of 98.1 (96.9–98.8). From the NHI database, we selected 31,471 patients with UC and randomly sampled age- and sex-matched controls in a 1:3 ratio (n = 91,638) with the absence of diagnostic codes of IBD (K51 for UC and K50 for CD). We excluded patients with previous cancer and missing data and selected only women in both the UC patient and control groups because our primary endpoint was the incidence of cervical cancer, which occurs only in the female genital tract.

This study was conducted in compliance with the tenets of the Declaration of Helsinki. We de-identified all identifiable personal information in the records before use to follow the privacy rules of the Health Insurance Portability and Accountability Act. The Institutional Review Board of Wonju Severance Christian Hospital approved this study (CR312044), and the need for informed patient consent was waived.

### Ascertainment of cervical cancer

The diagnosis of cervical cancer was defined according to the ICD-10 code C53. The Standardized incidence ratio (SIR) is calculated as the ratio of observed to expected cancer incidence. To calculate the expected incidence, we obtained information on cervical cancer incidence in patients with UC from the National Health Insurance (NHI). We then obtained the cancer incidence rate in the general population from the National Cancer Registry (NCR), which is a widely used source of official cancer data in Korea. Cancer incidence rates were similar between the NCR and NHI claims data because in South Korea, patients admitted to the hospital for cancer are considered incident cases. Incident cervical cancer cases were defined as those admitted to the hospital due to cervical cancer (ICD-10: C53). Patients with the V code 193–194, which represents specially registered cancer patients, were included to increase the accuracy of cancer diagnosis^9,10^. To improve the accuracy of the cancer diagnosis, patients with the V code 193–194, which represents specially registered cancer patients, were included. The V code system registers patients with rare and difficult-to-treat diseases, and is managed by the Korean NHI and Ministry of Health and Welfare.

### Statistical analysis

We calculated the SIR to determine whether patients with UC have a higher risk of cervical cancer than the general population. The incidence was defined as the number of observed cancer cases. The NCR provided the incidence rate of cancer in the general population at 5 years intervals from age 0 to 99 years. The number of expected cancer cases was calculated by multiplying the age-specific cancer incidence rate of the general population in 2011 (as reported by the NCR) by the person-years of patients with UC and cervical cancer. The 95% confidence intervals (CIs) for the SIR were calculated using the chi-square distribution for the number of observed cases from 1 to 9 and Byar’s approximation for 10 and above^11,12^. SIRs were calculated by using Microsoft Office Excel 2007 (Microsoft Corporation, Redmond, Washington DC, USA) and SAS program version 9.4 (SAS Institute Inc., Car, NC, USA).

Covariate variables for cervical cancer risk included comorbidities, socioeconomic status, residential area, and modified Charlson comorbidity index (CCI) score. The comorbidities (ICD-10 codes) considered were hypertension (I10–13, I15), diabetes mellitus (E78), cerebrovascular disease (I60–69), cardiovascular disease (I20–25, I34–37), and cholangitis (K83).

The CCI score was calculated by weighting the range of comorbidities based on ICD codes to predict 1 year mortality^13,14^. In our study, the CCI score was modified; tumor factors were excluded when calculating the CCI score as the primary endpoint of our study was determining cervical cancer risk. Patients who took the drug at least once were classified as drug users. Changes in drug types or doses over time and drug combinations were not considered.

Continuous variables are presented as means ± standard deviations, and categorical variables are presented as numbers and percentages. To compare characteristics between groups, Student’s t-test was used for continuous variables, and the chi-squared test was used for binary and categorical variables. Multivariate Cox regression models with Firth correction were used to assess the risk of cervical cancer in patients with UC, with reference to age-matched individuals. Hazard ratios (HRs) for cervical cancer were adjusted for age, sex, socioeconomic status, residential area, and comorbidities, including hypertension, diabetes mellitus, cholangitis, cerebrovascular disease, and cardiovascular disease.

Statistical analyses were two tailed, and a *p* value of < 0.05 was considered statistically significant. All analyses were performed using SAS program version 9.4 (SAS Institute, Inc.).

## Results

### Study population

**Figure 1:**
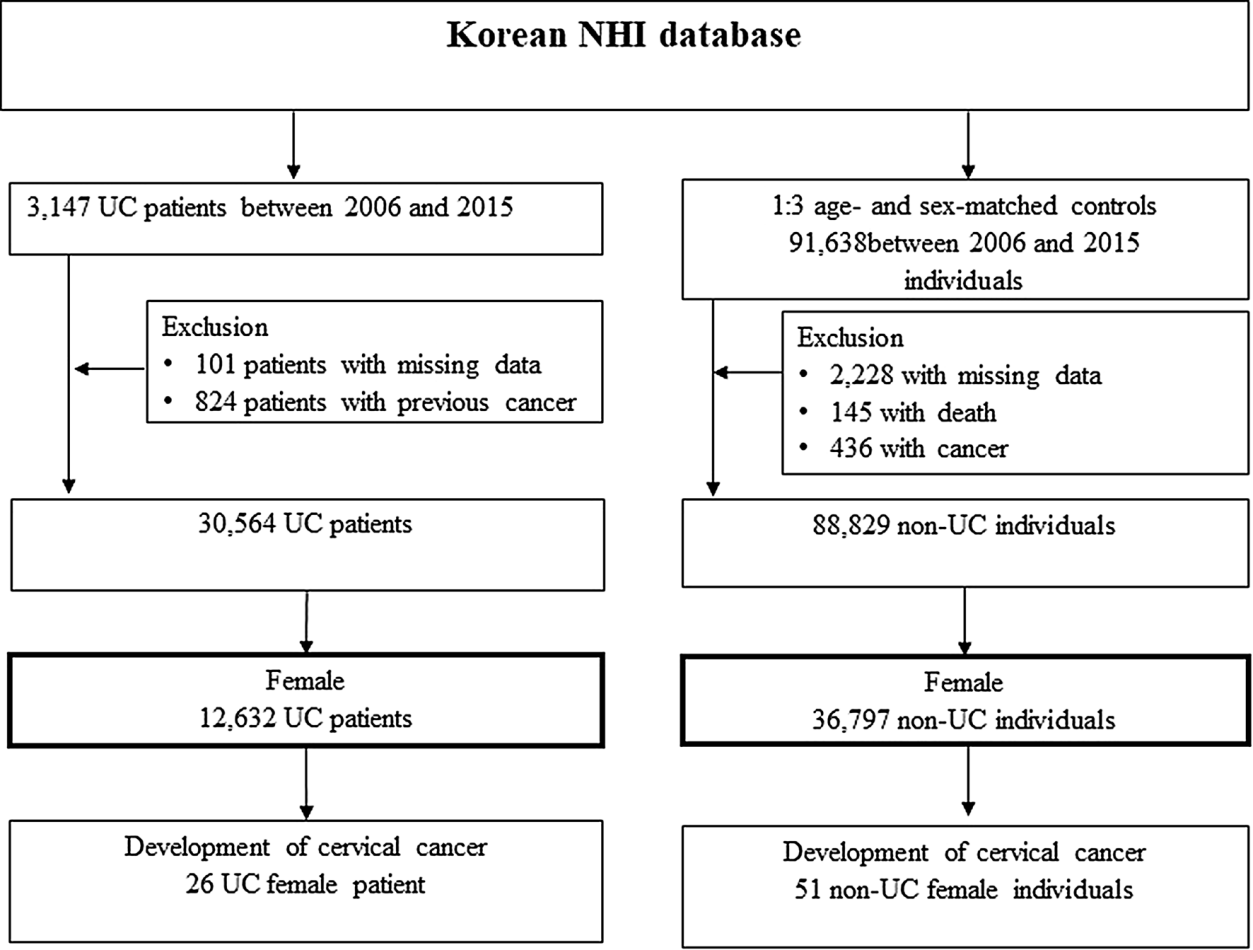
Flow chart of the study population.

A total of 31,471 patients were newly diagnosed with UC between 2006 and 2015 (Fig. 1). After excluding 925 patients, 30,546 patients with UC were enrolled in the study. Age- and sex-matched individuals from the general population numbered 91,638, of whom, 2,809 were excluded due to previous cancer, all-cause death, or discontinuation of insurance. In total, 88,829 matched individuals were included in the non-UC group. The UC and non-UC groups included 12,632 and 36,797 women, respectively, with equal proportions (41%). Only women from each group were included in the analysis. The median follow-up time was 5.31 ± 2.94 years (67,013 person-years) for the UC group and 5.40 ± 2.93 years (198,773 person-years) for the non-UC group.

The demographic and clinical baseline characteristics of the study population are presented in Table 1. The mean age of the UC group was slightly higher than that of the non-UC group (43.4 ± 16.5 vs. 43.1 ± 16.4 years). The UC group had higher proportions of high socioeconomic status, rural residential areas, and comorbidities including diabetes mellitus, cerebrovascular disease, cardiovascular disease, and cholangitis than the non-UC group.

**Table 1:** Baseline characteristics of the study population between 2006 and 2015.

Characteristic	UC patients	Non-UC individuals	*p* value
Total	12,632	36,797	
Person-year	67,013	198,773	
Age at diagnosis of UC*			
Mean ± SD	43.4 ± 16.5	43.1 ± 16.4	0.0301
< 40 years	5531 (43.8)	16,409 (44.6)	0.1149
≥ 40 years	7101 (56.2)	20,388 (55.4)	
Social economic status			< .0001
Low	3424 (27.1)	9811 (26.7)	
Mid	3694 (29.2)	12,944 (35.1)	
High	5514 (43.7)	14,042 (38.2)	
Residential area			< .0001
Urban	1003 (7.9)	3899 (10.6)	
Suburban	5612 (44.5)	15,699 (42.9)	
Rural	6006 (47.6)	17,034 (46.5)	
Comorbidities			
HTN	3322 (45.5)	9751 (50.9)	0.6583
DM	3102 (42.5)	8047 (42.0)	< .0001
Cerebrovascular disease	211 (2.9)	451 (2.4)	0.0002
Cardiovascular disease	198 (2.7)	400 (2.1)	< .0001
Cholangitis	287 (3.9)	363 (1.9)	< .0001
Modified CCI score**			< .0001
0	3374 (26.7)	11,385 (30.9)	
1	4276 (33.9)	12,494 (34.0)	
2	2593 (20.5)	6788 (18.4)	
≥ 3	2389 (18.9)	6130 (16.7)	
UC medication			
Steroid	5973 (28.4)	2,815 (97.5)	< .0001
5-ASA	12,505 (59.5)	10 (0.3)	< .0001
Thiopurines	1,947 (9.3)	48 (1.7)	< .0001
Biologics	601 (2.9)	14 (0.5)	< .0001
Cervical cancer	26 (0.21)	51 (0.14)	0.0983
Incidence rate***	38.8	25.7	
Follow-up period (years)	5.31 ± 2.94	5.40 ± 2.93	0.0014

Data are presented as the mean ± SD or number (%). CD, Crohn's disease; UC, ulcerative colitis; CCI, Charlson comorbidity index; HTN, hypertension; DM, diabetes mellitus; 5-ASA, 5-aminosalicylic acid.

*In non-UC Individuals, “age” was defined as the matched age to the UC patients when selected.

**CCI score without tumor factors.

***per 100,000 female per year.

### Incidence of cervical cancer in UC

During the study period, 26 (0.21%) among the 12,632 female UC patients developed cervical cancer, and 51 (0.14) among 36,797 female non-UC individuals developed cervical cancer (Table 1). The incidence of cervical cancer was 38.8 per 100,000 women per year in female UC patients and 25.7 per 100,000 women per year in female controls, respectively. The SIR (95% CI) of cervical cancer in the UC and non-UC groups were 2.04 (1.33–2.98) and 1.35 (1.00–1.77), respectively (Table 2).

**Table 2:** Standardized incidence ratio of cervical cancer in patients with ulcerative colitis.

	UC	Non-UC individuals
Observed, N	26	51
Expected, N	12.77	37.79
SIR (95% CI)	2.04 (1.33–2.98)	1.35 (1.00–1.77)

*SIR* standardized incidence ratio, *CI* confidence interval.

### Risk of cervical cancer in patients with UC

The adjusted HR (95% CI) for cervical cancer in the UC group, compared to the non-UC group, was 1.56 (0.97–2.50). When stratified by age groups and compared to the non-UC group, the adjusted HRs (95% CIs) for cervical cancer in the UC group were 9.89 (0.06–1653.78) in 0–19 years, 1.62 (0.65–4.02) in 20–39 years, 0.79 (0.35–1.79) in 40–59 years, and 3.65 (1.54–8.66) in ≥ 60 years (Table 3).

**Table 3:** Hazard ratio of ulcerative colitis for cervical cancer.

	UC			
	Cervical cancer cases	Adjusted HR	95% CI	*p* value
Overall	26	1.56	0.97–2.50	0.0667
Age (years)				
0–19	1	9.89	0.06–1653.78	0.3804
20–39	7	1.62	0.65–4.02	0.3002
40–59	7	0.79	0.35–1.79	0.5690
≥ 60	11	3.65	1.54–8.66	0.0033

Multiple analysis including age, sex, social economic status, Residential area, hypertension, diabetes mellitus, cerebrovascular disease, cardiovascular disease, and cholangitis. Reference: matched controls of UC. *HR* hazard ratio.

### Risk factors for cervical cancer within patients with UC

The UC group was divided into two groups, cervical and non-cervical cancer groups, to explore the risk factors associated with cervical cancer in patients with UC (Table 4). The cervical cancer subgroup had a higher age at diagnosis (53.19 ± 18.3 vs. 43.4 ± 16.52 years), a greater proportion of individuals with low socioeconomic status (13 (50%) vs. 2259 (27.1%)), and more rural residents (5 (19.2%) vs. 998 (7.9%)) than the non-cervical cancer subgroup. Multivariate analysis did not reveal any independent risk factors for cervical cancer in UC patients (Table 5). The statistical model included age at UC diagnosis, sex, socioeconomic status, Residential area, hypertension, diabetes mellitus, cerebrovascular disease, cardiovascular disease, and cholangitis (Table 5). Among patients with UC, age ≥ 40 years and living in rural residential area were potential risk factors for cervical cancer; however, these findings were not statistically significant (HRs (95% CIs): 2.18 (0.83–5.35) for age ≥ 40 years, 2.38 (0.86–6.62) for rural residential area, respectively). High socioeconomic status was significantly associated with a HR than low socioeconomic status (HR, 0.29; 95% CI 0.11–0.76), but this finding was not statistically significant.

**Table 4:** Baseline characteristics of patients with ulcerative colitis.

Characteristic	Ulcerative colitis		
	Cervical cancer	Non-cervical cancer	*p* value
Total	26	12,606	
Person-year	68	66,945	
Age at diagnosis of UC			
Mean ± SD	53.19 ± 18.3	43.4 ± 16.52	0.0025
0–19 years	1 (3.8)	779 (6.2)	0.0149
20–39 years	7 (26.9)	4 744 (37.6)	
40–59 years	7 (26.9)	4 824 (38.3)	
≥ 60 years	11 (42.3)	2 259 (17.9)	
Socioeconomic status			
Low	13 (50)	3 411 (27.1)	0.0222
Mid	7 (26.9)	3 687 (29.2)	
High	6 (23.1)	5 508 (43.7)	
Residential area			
Urban	13 (50)	5 993 (47.6)	0.0705
Sub-urban	8 (30.8)	5 604 (44.5)	
Rural	5 (19.2)	998 (7.9)	
Comorbidities			
HTN	6 (54.5)	3 316 (45.4)	0.8262*
DM	4 (36.4)	3 098 (42.5)	0.3642*
Cerebral vascular disease	1 (10.1)	210 (2.9)	0.3549*
Cardiovascular disease	0	198 (2.7)	1.0000*
Cholangitis	0	287 (3.9)	1.0000*
CCI without tumor factor			
0	8 (30.8)	3 366 (26.7)	0.4281
1	5 (19.2)	4 271 (33.9)	
2	6 (23.1)	2 587 (20.5)	
≥ 3	7 (26.9)	2 382 (18.9)	
UC medication			
Steroid	10 (25.6)	5 963 (28.4)	0.3670
5-ASA	25 (64.1)	12 480 (59.5)	0.2312*
Thiopurines	3 (7.7)	1 944 (9.3)	0.7875*
Biologics	1 (2.6)	600 (2.9)	1.0000*
Follow-up period, year	2.62 ± 2.28	5.31 ± 2.94	< .0001

Data are presented as the mean ± SD or number (%). *p* values with * were obtained using a Fisher exact test.

**Table 5:** Risk factors of cervical cancer in patients with ulcerative colitis.

	Ulcerative colitis		
	HR	95% CI	*p* value
Age at diagnosis of UC			
< 40	1		
≥ 40	2.18	0.83–5.35	0.091
Social economic status			
Low	1		
Mid	0.53	0.21–1.32	0.173
High	0.29	0.11–0.76	0.012
Residential area			
Urban	1		
Suburban	0.68	0.28–1.64	0.388
Rural	2.38	0.86–6.62	0.097
Comorbidities			
HTN	0.70	0.26–1.91	0.487
DM	0.47	0.16–1.39	0.170
Cerebral vascular disease	3.99	0.73–21.69	0.110
Cardiovascular disease	0.80	0.05–14.11	0.878
Cholangitis	0.79	0.05–13.45	0.872

Multiple analyses including age, sex, socioeconomic status, Residential area, hypertension, diabetes mellitus, cerebrovascular disease, cardiovascular disease, and cholangitis for UC.

## Discussion

In this study, we found that the incidence rate of cervical cancer in the UC group was higher than that in the non-UC group (38.8 per 100,000 female per year vs. 25.7 per 100,000 female per year). The SIR of cervical cancer in UC patients was also higher than that in the general population (SIR 2.04; 95% CI 1.33–2.98). The HR for cervical cancer at overall age was calculated as 1.56 (95% CI 0.97–2.50) in UC group without statistical significance. In subgroup analysis, the HR in elderly UC patients (≥ 60 years) was 3.65 (95% CI 1.54–8.66) for cervical cancer as compared to elderly non-UC individuals (≥ 60 years).

UC is a rare condition. However, it is crucial for healthcare providers to provide appropriate prevention methods or screening for co-morbidities to UC patients. We focused on the incidence of cervical cancer in female UC patients, but not that in other systemic autoimmune diseases, such as rheumatoid arthritis and systemic lupus erythematosus.

During the period of 2006–2015, according to the Korean Central Cancer Registry, cervical cancer was the fifth most common cancer among women in South Korea, and the incidence rate of cervical cancer in South Korea was relatively high with an age-standardized incidence rate of 9.9 per 100,000 women in 2006 and 8.7 per 100,000 women in 2015. According to a report by the National Cancer Center, the screening rate for women aged 20–69 increased from 37.9% in 2006 to 57.3% in 2015^15^.

Cervical cancer screening guidelines in South Korea recommend regular screening for all sexually active women aged 20 to 69 years. The Pap test is recommended for women aged 20 to 29 years and is performed every two years. Women aged 30 to 69 years are recommended to have both the Pap test and HPV test every three years. If both tests are negative, then the screening interval can be extended to every five years. Women who have abnormal test results may require further diagnostic tests and treatments^16,17^.

During the period from 2006 to 2015, there was a significant increase in the cervical cancer screening rate in South Korea. According to a study using Korean National Cancer Screening Survey data, the screening rate for women increased from 68.0% in 2006 to 76.2% in 2015^18^. In South Korea, the HPV vaccination was included in the national immunization program in 2016 for girls aged 12–13 years old. According to a report by the Korean Centers for Disease Control and Prevention (KCDC), the HPV vaccination rates for girls aged 12–13 were 56.4% in 2018, and 63.8% in 2019^19^.

In our study, the increased risk of cervical cancer in elderly UC patients (≥ 60) may be attributed to the low HPV vaccination and cervical screening rates in this age group. However, since our data did not include information on cervical cancer screening or HPV vaccination history, it was difficult to associate these factors with the increased risk of cervical cancer in UC patients over the age of 60.

UC patients have defects in both innate and adaptive immune systems, including T-cell defects^20^. T-cell function impairment hinders the attraction of professional antigen-presenting cells, which may cause problems in triggering anti-HPV immune responses. Therefore, we suggest that aside from immunosuppressive drugs, UC itself can increase the risk of cervical cancer caused by persistent HPV infection.

These results are consistent with previous studies suggesting an increased risk of cervical cancer in UC patients^1–3,21,22^. However, no statistically significant results were obtained except in the study by Jung et al.^3^. Several studies have shown that the risk of cervical neoplasia increases in IBD patients when cervical intraepithelial dysplasia (CIN) and cervical cancer are analyzed together^5,23^. In a Denmark cohort study, women with IBD had an increased risk of high-grade dysplasia and cervical cancer [CIN2+] (IRR 1.66, 95% CI 1.21–2.25) and persistent or recurrent CIN during follow-up (odds ratio 1.89, 95% CI 1.06–3.38)^23^. However, when analyzing the risk of cervical cancer alone in IBD, the risk did not increase^23^. The reason is thought to be that the total number of women with IBD is as small as 2068, and the number of cervical cancer cases is smaller still with only two cases^23^.

Although previous studies have often evaluated the risk of cervical cancer only in patients with IBD using immunosuppressants, the effect of immunosuppressant use on cervical cancer development in each study is controversial. Some studies have shown that immunosuppressants do not significantly contribute to cervical abnormalities in patients with IBD^21,23–25^. However, other studies confirmed that cervical abnormalities increased in patients with IBD using immunosuppressive drugs^26,27^, and the risk of high-grade dysplasia and cervical cancer increased in IBD patients using immunosuppressants^28^.

HPV infection is associated with progression of precancerous lesions of the cervix into cervical cancers, and this process often takes long time. The mean time from HPV infection to diagnosis of CIN3 is 9.4 ± 4.1 years and progression from CIN3 to invasive cervical cancer takes 10–20 years, depending on genotype^29,30^. A recent modelling study found that the median time from acquisition of HPV to cancer detection ranged from 17.5 to 26.0 years^31^. Cervical cancer can be prevented through screening by identifying and treating the precancerous lesions. Usually HPV-screening every 5 years is enough to prevent cervical cancer. However, one population-based study revealed that women with IBD are less likely to have Papanicolaou (Pap) tests, and approximately 54% of them had regular Pap tests^32^.

Cervical cancer screening is typically recommended to start at age 25 because the risk of developing cervical cancer is low in women under 25 years old. Cervical screening among women under age 25 years has minimal impact on rates of carcinoma, but it leads to overdiagnosis or overtreatment of precancerous lesions that are destined to regress or not progress to cervical cancer^33^. The American Cancer Society (ACS) recommends that women initiate cervical cancer screening at the age of 25 years, and undergo primary HPV testing every five years until the age of 65 years^34^. The American College of Obstetricians and Gynecologists recommends not stopping cervical cancer screening for HIV-infected patients, even if they are over the age of 65^35^. In Korea’s national cancer screening program, women aged 20 and over are eligible for cervical cancer screening, which is provided every two years^17^.

Our study showed that elderly-onset UC was a risk factor for cervical cancer, and cervical cancer occurred not only at the time of UC diagnosis but also during the follow-up period. Therefore, in patients with elderly-onset UC, it is important to perform appropriate cervical cancer screening at the time of UC diagnosis and continue cervical cancer screening beyond the age at which it is recommended to stop cervical cancer screening.

HPV infection persists only in a small portion, and only 10% of carriers develop precancerous lesions^36^. The risk of CIN 3 or cervical cancer in women with HPV infection varies depending on the type of HPV^37^. HPV 16, 18, 31, 33 and 45 infections are associated with risk of progression to CIN 3 or cervical cancer^37–39^. Spontaneous regression occurs at a significant rate^40,41^. In the case of CIN 3, invasive cancer occurred in only 30.1% of cases when follow-up was performed for 30 years^42^. Therefore, not all cervical dysplasia results in cervical cancer. Because the incidence of cervical cancer is low, most studies have analyzed the risk of cervical dysplasia and cancer. In one case-controlled cohort study, cervical cancer developed in only two patients in the IBD group; thus, they included CIN2 and CIN3^23^. However, in our study, since the number of cervical cancer cases was sufficient (26 and 51 in the UC and non-UC groups, respectively), it was possible to analyze the statistically significant increase in the risk of cervical cancer with UC. For the reasons mentioned above, we did not include CIN in our study.

There is evidence to suggest that living in certain residential areas may be associated with an increased risk of developing UC. Several studies have found that people living in urban areas or developed countries are more likely to develop UC compared to those living in rural areas or developing countries^43,44^. One possible explanation for this association is that exposure to environmental factors, such as air pollution or a Western-style diet, may contribute to the development of UC. We considered residential area as a confounding factor to be controlled in our study.

Our study has several strengths. Firstly, it is the first to investigate the effects of age on cervical cancer in patients with UC. Stratification based on age at diagnosis revealed a significant increase in cervical cancer risk for UC patients aged 60 years or older, emphasizing the need for cervical cancer screening in this population. Additionally, our study is the second population-based study to evaluate cervical cancer risk in Asian patients with UC who have distinct epidemiological and genetic profiles compared to their Western patients^45^. The only previous study in Korea had a shorter median follow-up time of 2.20 years for UC patients, which may have overestimated the risk of cervical cancer due to frequent health checkups^3^. In contrast, our study had a longer median follow-up time of 5.31 ± 2.94 years. Furthermore, our study focused solely on UC patients, allowing us to analyze the increased risk of cervical cancer specifically in this population.

However, our study had several limitations. First, CIN is an abnormal growth of cells on the cervical surface and is considered a precursor lesion of cervical cancer. Including CIN will increase the number of study participants compared to cervical cancer alone. However, we did not include CIN in our research. Second, there was no information on possible confounding factors, such as smoking, combination oral contraceptive use, and multiple sexual partners, known to be related to an increased risk of cervical cancer^36^. In addition, there is no information on the status and onset time of HPV infection, HPV vaccination, and cervical cancer screening. Since the prophylactic effect of the HPV vaccination on cervical cancer and its precursor lesions has been proven^46^, it is also important to know whether a vaccination is available; however, there is no information available about this. Third, because we used administrative data, we could not adjust for the extent and severity of UC. Finally, we were unable to reveal a relationship between the use of immunomodulatory medications and the development of cervical cancer due to the low incidence of cervical cancer in women with IBD receiving immunomodulatory medications.

## Conclusions

While the standardized incidence ratio (SIR) of cervical cancer was higher among UC patients (SIR 2.04; 95% CI 1.33–2.98), the adjusted hazard ratio (HR) for cervical cancer in UC patients was not statistically significant compared to non-UC controls (adjusted HR 1.56, 95% CI 0.97–2.50). However, when analyzing by age group, the adjusted HR for cervical cancer in elderly UC patients (≥ 60 years) was significantly higher than in elderly non-UC controls (≥ 60 years). Therefore, it is recommended that physicians review the cervical cancer screening history of elderly UC patients and advise them to undergo cervical cancer screening if there is no adequate screening history.

## Data Availability

The data that support the findings of this study are available from Korean National Health Insurance but restrictions apply to the availability of these data, which were used under license for the current study, and so are not publicly available. However, data are available from the authors upon reasonable request and with permission of National Health Insurance.
